# Diagnosis of *FOXG1* syndrome caused by recurrent balanced chromosomal rearrangements: case study and literature review

**DOI:** 10.1186/s13039-020-00506-1

**Published:** 2020-09-03

**Authors:** Connor P. Craig, Emily Calamaro, Chin-To Fong, Anwar M. Iqbal, Alexander R. Paciorkowski, Bin Zhang

**Affiliations:** 1 Department of Pathology and Laboratory Medicine, University of Rochester Medical Center, 601 Elmwood Ave, Box 608, Rochester, NY 14642 USA; 2 School of Medicine and Dentistry, University of Rochester, 601 Elmwood Ave, Rochester, NY 14642 USA; 3 Department of Pediatrics, University of Rochester Medical Center, 601 Elmwood Ave, Rochester, NY 14642 USA; 4 Department of Neurology, University of Rochester Medical Center, 601 Elmwood Ave, Rochester, NY 14642 USA; 5 Center for Neural Development and Disease, University of Rochester Medical Center, 601 Elmwood Ave, Rochester, NY 14642 USA; 6 Departments of Neuroscience and Biomedical Genetics, University of Rochester Medical Center, 601 Elmwood Ave, Rochester, NY 14642 USA

**Keywords:** FOXG1, Haploinsufficiency, Postnatal microcephaly, FISH, Enhancer, Chromosomal rearrangement, Diagnosis

## Abstract

**Background:**

The *FOXG1* gene plays a vital role in mammalian brain differentiation and development. Intra- and intergenic mutations resulting in loss of function or altered expression of the *FOXG1* gene cause *FOXG1* syndrome. The hallmarks of this syndrome are severe developmental delay with absent verbal language, post-natal growth restriction, post-natal microcephaly, and a recognizable movement disorder characterized by chorea and dystonia.

**Case presentation:**

Here we describe a case of a 7-year-old male patient found to have a de novo balanced translocation between chromosome 3 at band 3q14.1 and chromosome 14 at band 14q12 via G-banding chromosome and Fluorescence In Situ Hybridization (FISH) analyses. This rearrangement disrupts the proximity of *FOXG1* to a previously described smallest region of deletion overlap (SRO), likely resulting in haploinsufficiency.

**Conclusions:**

This case adds to the growing body of literature implicating chromosomal structural variants in the manifestation of this disorder and highlights the vital role of cis-acting regulatory elements in the normal expression of this gene. Finally, we propose a protocol for reflex FISH analysis to improve diagnostic efficiency for patients with suspected *FOXG1* syndrome.

## Introduction

The Forkhead Box G1 (*FOXG1*) gene [OMIM: 164874], located on chromosome 14q12, encodes the protein forkhead box protein G1 (FOXG1). It belongs to a class of winged-helix transcriptional regulators and contains a highly conserved fork head DNA-binding domain. This protein plays an important role in mammalian brain development, with high levels of expression in the developing fetal telencephalon [1–4]. Specifically, it is expressed in the rostral forebrain prior to differentiation into the telencephalon and diencephalon, indicating its role in early differentiation between these structures [5]. It exerts its effects via DNA binding-dependent and -independent mechanisms to encourage neocortical progenitor proliferation and prevent precocious differentiation [6]. In addition to regulating neocortical progenitor cell populations, it also plays an important role in controlling post-mitotic pyramidal cortical neuron migration and post-migration dorsal–ventral patterning to establish normal cortical laminar structure and the corpus callosum [7].

Both intra- or inter-genic mutations resulting in altered *FOXG1* expression or protein function causes *FOXG1* syndrome. The syndrome is characterized by post-natal growth deficiency, postnatal microcephaly, intellectual disability, restricted language development, autism-like social deficits, stereotypies and dyskinesias, epilepsy, poor sleep, irritability, excessive crying episodes, recurrent aspiration, and gastroesophageal reflux [8]. Characteristic findings on imaging include: frontal predominant simplified gyral patterning, reduced white matter volume, and callosal hypogenesis [8]. The course of epilepsy in patients with *FOXG1* syndrome varies based on the underlying genetic mutation. Those with deletions and intragenic mutations tend to have a wide variety of seizure types, ranging from complex partial to generalized tonic–clonic, while those with duplications frequently exhibit infantile spasms [9]. Clinically, the developmental encephalopathy index (DEI) has proven useful in delineating *MECP2* and *FOXG1* syndrome, showing that those with *FOXG1* syndrome had greater impairment overall, with significantly worse function in the domains of fine motor skills, receptive language, reciprocity and ability to walk [10].

Different mutation mechanisms, including intragenic point or indel (insertions and deletions) mutations, microdeletions, or balanced chromosomal rearrangements can lead to *FOXG1* haploinsufficiency resulting in *FOXG1* syndrome. This makes efficient genetic diagnosis of the disease quite challenging [11–14]. Here we report a case of *FOXG1* syndrome in a 7-year-old Puerto Rican male patient found to have a de novo balanced chromosomal rearrangement with the breakpoint in an intergenic region between *FOXG1* and a nearby smallest critical enhancer region (SRO). This report shows that chromosomal rearrangements disrupting a distant regulatory enhancer of *FOXG1* is a recurrent event, and that a follow-up FISH analysis is important to reach a definitive genetic diagnosis for patients with balanced rearrangements involving 14q11-q13 with *FOXG1* syndrome in the differential diagnosis.

## Clinical report

The patient is a 7-year-old male born in Puerto Rico at 33-week gestation to an 18-year-old gravida 1 para 0 female of Puerto Rican descent. No prenatal complications or exposures are reported in the available medical record. Birth weight was 1871 g (35th percentile; standard deviations (SD) − 0.38) and length was 48 cm (93rd percentile; SD 1.82). Head circumference at birth is not available in the medical record. Newborn screening was reportedly normal. The patient was admitted to the neonatal intensive care unit for poor weight gain and jaundice requiring phototherapy and discharged after 8 days.

At approximately 4 months of age the patient’s parents noted developmental motor delays with an inability to raise his head, push himself up, and limited overall movement as well as microcephaly. An initial work-up at the time was notable for: metabolic screening showed non-specific elevations in glutamate and mild abnormalities in excretion of 4-hydroxyphenylacetate, mildly elevated NH_3_, and elevated lactate at two to three times the upper reference limit. Magnetic resonance imaging (MRI, unavailable for review) was reportedly unremarkable. Cytogenetic studies showed a balanced (3;14) translocation.

The patient had no speech development at 1 year of age. Seizures began at 18 months and were characterized by staring episodes which would progress to full-body limpness, stiffening, followed by jerking movements. The patient had five seizures prior to control with levetiracetam.

At 3 years of age the patient’s mother moved with him to the United States where evaluation showed a severely underweight child (12.08 kg; 5th percentile; SD − 1.61) with microcephaly (head circumference 42.9 cm; SD − 4.0), congenital esotropia, reduced muscle bulk, decreased axial and increased appendicular tone with antigravity strength throughout. The patient remained non-verbal but exhibited social smiling, and the ability to track objects. The patient had poor weight gain due to severe oropharyngeal dysphagia with silent aspiration on pharyngogram, eventually necessitating G-tube placement.

EEG performed at 4 years of age showed a disordered background with slower than expected posterior dominant rhythm consistent with a mild diffuse encephalopathy. There were no focal or epileptiform changes noted.

At present the patient continues to have severe global developmental delay, with no speech development, but he attempts to make sounds. He is able to roll over and grab onto objects. He continues to exhibit upper and lower extremity spasticity with grossly ataxic movements, and excessive purposeless arm movements. His seizures continue to be well-controlled. He has recently displayed episodes of inappropriate laughing and crying.

## Methods

### G-banding chromosome and FISH analysis

**Fig. 1:**
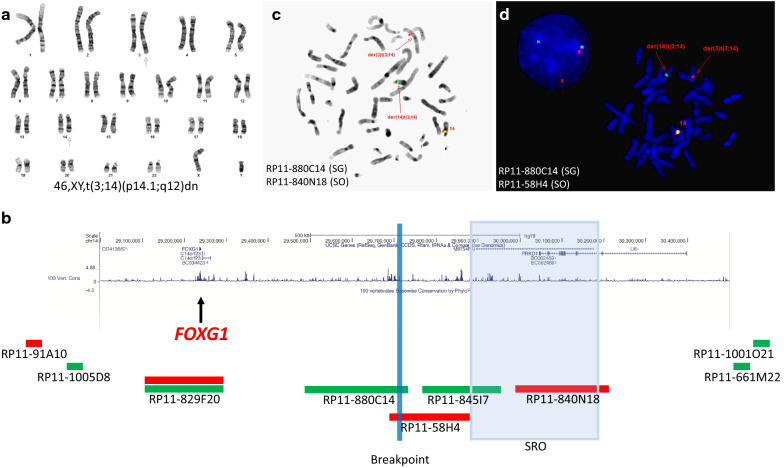
Cytogenetic Analysis. **a** Chromosome G-banding analysis identified an apparently balanced de novo translocation involving chromosome 3 and chromosome 14 in the proband. **b** Selected BAC clones on chromosome 14 were fluorescent-dye labeled as indicated. Five probes within the interval of chr14:29,000,000-30,500,000 are scaled in terms of their size and location, while four peripheral ones are not. **c** and **d** Fluorescent in situ hybridization (FISH) analysis of metaphase chromosomes reveals the breakpoint indicated by a blue line at approximately chr14:29,689,627-29,728,030 between the *FOXG1* gene (chr14:29,236,278-29,239,483) and the previous defined smallest region of deletion overlap(SRO, chr14:29,875,672-30,173,942). **c**, inverted DAPI staining; **d**, DAPI staining. SG, spectrum green; SO, spectrum orange. dn, de novo

Peripheral blood samples were cultured using standard cytogenetic methods for 72 h with phytohemagglutinin (PHA) stimulation. Chromosomes were analyzed by G-banding using trypsin digestion and Wright’s staining (GTW). Twenty metaphase spreads were analyzed. The karyotypes were described according to An International System for Human Cytogenetic Nomenclature (ISCN 2016). Fluorescent In Situ Hybridization (FISH) analyses were performed with standard techniques using bacterial artificial chromosome (BAC) probes (Empire Genomics, NY; listed in Fig. 1d). All genomic coordinates are based on the Human GRCh37/hg19 Genome Assembly.

### Comparative genomic hybridization (CGH)

DNA was extracted from the patient’s peripheral blood using QIAamp^®^ DNA Blood Mini Kit (Qiagen, CA). A Nanodrop ND-1000 spectrometer (Thermo Scientific, DE) was used for determination of DNA concentrations. Microarray experiments were performed using the SurePrint G3 Human CGH Microarray 4 × 180 K platform (Agilent Technologies, CA). Data were analyzed and visualized using the Agilent CytoGenomics 4.0 software (Agilent Technologies, CA). Commercially available pooled male DNA (Promega, WI) was used as control DNA. Briefly, after the initial DNA denaturation step, the patient’s DNA and control DNA (500 ng) were labeled with dyes Cyanine-5dUTP and Cyanine-3dUTP, respectively, by using Agilent’s Universal Linkage System (ULS™) technology as per the manufacturer’s recommendations. The hybridization and subsequent steps were performed as per the manufacturer’s recommendations (Agilent Technologies, CA). The slide was scanned in Agilent’s high-resolution Model #G2505C scanner at 3 µm resolution. The scanned image file was directly imported to the Agilent CytogGenomics 4.0 software for the visualization and analysis.

## Results

Chromosome analysis identified a balanced reciprocal translocation involving breakpoints on the short arm of chromosome 3 at band 3p13 and the long arm of chromosome 14 at band 14q11.2 (Fig. 1a). The karyotype was defined as 46,XY,t(3;14)(p13;q11.2). Chromosome analysis on peripheral blood of both parents revealed normal karyotypes, indicating that the identified translocation in the proband is de novo. Chromosomal rearrangement has been shown to cause disruption or dysregulation of a gene (or genes) near the breakpoint, so detailed breakpoint characterization has been beneficial to identify and annotate genes important in human development [15]. In addition, such chromosomal rearrangements may be more complex when analyzed at a higher level of resolution, and are associated with deletions around breakpoints in 37% cases [15]. Microarray analysis revealed this proband carries no clinically significant genomic imbalances and no identifiable aberrations in the proximity of breakpoints (data not shown). We hypothesize that the de novo balanced (3;14) translocation is likely pathogenic and elected to map the breakpoints using FISH analysis.

FISH analysis using a series of bacteria artificial chromosome (BAC) probes (RP11-905F6, RP11-689N19, RP11-844M13 on chromosome 3, and RP11-91A10, RP11-1001O21, RP11-1005D8 and RP11-661M22 on chromosome 14) defined the breakpoint within a 2.7 Mb interval of chr3:66,441,621-69,183,895 at band 3p14.1, and the breakpoint within a 5.9 Mb interval of chr14:24,994,466-30,870,173 within band 14q12 (data not shown). The karyotype was therefore refined as 46,XY,t(3;14)(p14.1;q12)dn. Genes within these regions were investigated in detail by literature search and genotype–phenotype comparison. There are 10 protein-coding genes, including *EOGT* (OMIM#614789) and *LMOD3* (OMIM#616112) within the chr3 breakpoint interval and 7 refseq protein-coding genes, including *FOXG1* (OMIM#164874) and *PRKD1* (OMIM#605435) within the chr14 breakpoint interval. *EOGT* is associated with autosomal recessive Adams-Oliver syndrome 4, and *LMOD3* with autosomal recessive Nemaline myopathy 10. *FOXG1* is associated with autosomal dominant *FOXG1* syndrome, and *PRKD1* with autosomal dominant congenital heart defects and ectodermal dysplasia. Haploinsufficiency of *FOXG1* caused by long-range position effects of intergenic structural variants is one underlying molecular mechanism causing *FOXG1* syndrome [11]. As the proband presented a clinical phenotype similar to *FOXG1* syndrome (discussed below), we focused on the fine mapping of the breakpoint on chromosome 14. BAC probe (RP11-829F20), which covers the entire *FOXG1* gene, was selected and co-hybridized along with the RP11-1001O21 BAC probe, and revealed the breakpoint is distal to the *FOXG1* gene (the gene itself is likely not disrupted), and within a 1.6 Mb interval of chr14:29,303,506-30,870,173. In order to further define the breakpoint relative to the *FOXG1* gene, we selected four additional BAC probes (Fig. 1b). The RP11-829F20 and RP11-880C14 probes label the derivative chromosome 14, while the RP11-58H4, RP11-845I7, and RP11-840N18 probes stain the derivative chromosome 3 (Fig. 1c, d). These findings indicate the breakpoint occurred in an intergenic ~ 38 Kb region (approximately around chr14:29,689,627- 29,728,030) between the *FOXG1* gene (~ 450 Kb proximal) and the smallest region of deletion overlap previously defined (SRO, ~ 148 Kb distal) [11]. In conclusion, metaphase FISH analysis using two BAC probes of RP11-829F20 (*FOXG1*) and RP11-840N18 (*SRO*) defined the breakpoint for this case, and can in principle diagnose 16/16 reported *FOXG1* syndrome cases which present structural variants involving chromosome 14 [11].

## Discussion

*FOXG1* syndrome results from intragenic or intergenic mutations resulting in haploinsufficiency of *FOXG1*. *FOXG1* syndrome has a well-defined clinical phenotype characterized in numerous case reports and cohort studies as postnatal growth restriction, post-natal microcephaly, global developmental delay with absence of language, movement disorder characterized by chorea and dystonia, deficient social reciprocity, variable forms of epilepsy, poor sleep patterns, paroxysmal laughter/crying, recurrent aspiration, with common neuroimaging findings of simplified gyral patterning reduced white matter frontal lobe volume, hypogenesis of the corpus callosum, and delayed myelination [8, 16–18].

Here we report a case of developmental encephalopathy in a 7 year-old-male patient with microcephaly, global developmental delay, seizures, appendicular spasticity with decreased axial tone, stereotypies, and inappropriate laughing/screaming spells. From the available medical record, microcephaly was not noted until approximately 4 months of age, though a final determination of whether it is truly post-natal microcephaly is not possible. An MRI was performed and reportedly was normal, without agenesis or malformation of the corpus callosum, but the images were not available for our review. Cytogenetic analysis using chromosome, microarray, and FISH demonstrated a de novo balanced translocation between chromosome 3 at band 3q14.1 and chromosome 14 at band 14q12. The breakpoint on chromosome 14 involves an intergenic region approximately 450 kb distal to the *FOXG1* gene.

Overall the patient’s phenotype as described above is consistent with a developmental encephalopathy, and cytogenetic analysis confirms a diagnosis of *FOXG1* syndrome [10]. It should be noted, however, that medical records are not available for the patients first 3 years of life, and much of the history from that time period was obtained via the patient’s family. This represents a potential weakness of this paper, and highlights the difficulty of using diagnostic criteria reliant on regular healthcare access and detailed neonatal and pediatric records.

This case is notable due to its unique cytogenetics. The translocation described does not disrupt the *FOXG1* gene itself, but rather its proximity to an enhancer element. This structural variant likely disrupts the normal *cis*-acting regulatory elements typically responsible for controlling *FOXG1* expression, resulting in effective haploinsufficiency. Chromosomal structural variants resulting in a “congenital variant of Rett syndrome” have been previously described, as well as cases of deletions resulting in the disruption of *FOXG1* regulatory elements [19–21]. Other reports have found examples of chromosomal structural variants disrupting *FOXG1* regulatory elements [8, 13, 22–24]. Mehrjouy et al. published one of the more comprehensive investigations into regulatory variants in *FOXG1* syndrome, examining the role of topologically associated domains and long range positional effects on *FOXG1* expression [11]. They defined a smallest region of deletion overlap (SRO) of approximately 430 kb located over 600 kb 3′ to *FOXG1*. They conclude that structural variants resulting in disruption of regulatory elements within the SRO results in the *FOXG1* phenotype. In our case, the breakpoint event (~ 450 Kb distal to *FOXG1*) also occurs between *FOXG1* and the SRO region, presumably resulting in loss of *FOXG1* expression and a disease phenotype. This supports the idea that such disease-causing chromosomal rearrangements disrupting a distant regulatory enhancer are recurrent in *FOXG1* syndrome. A unique genomic/chromatin characteristic at this locus may predispose it to breakage and rearrangement, warranting further molecular investigation.

A genotype–phenotype correlation study has also been performed in a cohort of 83 patients with *FOXG1* intragenic variants and revealed high phenotypic variability. Mild cases were associated with missense variants in the forkhead conserved site 1, severe phenotypes with truncating variants, and the most severe cases with the N-terminal truncating mutations [17]. We summarized the clinical features associated with different *FOXG1* variants, including *FOXG1* intragenic mutations [17], balanced translocations and microdeletions resulting in enhancer disruption [11], and microduplications associated with increased gene dosage [11] (Table 1). For intragenic mutations, we listed the most severe group with N-terminal truncating variants and the mildest group with missense variants within the forkhead conserved site 1 [17]. It is interesting that structural variants with a presumable enhancer disruption appeared to show higher severity than the truncating group, while the microduplication group showed milder phenotypes than the missense group (Table 1). The phenotype observed in this study is consistent with the severe phenotype associated with an enhancer disruption, despite our ability to accurately assess anomalies of the corpus collosum (Table 1). This suggests that the enhancer disruption may decrease gene expression of the *FOXG1* gene during development to a level of a complete gene deletion, resulting in a null allele and a severe haploinsufficient phenotype. We suspect that the defined enhancer may act as a super enhancer and regulate more genes within its proximity. Haploinsufficiency of adjacent genes could contribute to the phenotypic severity observed in this group. More detailed molecular characterization of the enhancer disruption will help better characterize this genotype–phenotype association.

**Table 1:** Clinical features associated with different FOXG1 variants

	*FOXG1* intragenic missense mutations [17]	*FOXG1* intragenic truncating mutations [17]	Balanced translocations or microdeletions [11]	Microduplications involving *FOXG1* [11]	Balanced Translocation (this study)
	Forkhead cs1 missense	N-terminal nonsense or frameshift	Enhancer disruption	Increased dosage	Enhancer disruption
Sample size of each study	12	37	16	9	1
Genetic test	Sequencing	Sequencing	Karyotyping and FISH^a^	Array and FISH	Karyotyping and FISH^a^
Severe intellectual disability	100%; 12/12	100%; 37/37	100%; 11/11	10%; 1/9	+
HC at follow-up (< − 2 SD)	50%; 5/10	96%; 23/24	100%; 11/11 (postnatal microcephaly)	22%; 2/9 (postnatal microcephaly)	+
HC at birth (< 2 SD)	13%; 1/8	33%; 6/18	NA	NA	NA
No walking (absent)	33%; 4/12	91%; 31/34	100%; 11/11	0%; 0/9	+
No verbal speech	67%; 8/12	86%; 30/35	100%; 11/11	78%; 7/9	+
Social interaction	13%; 1/8 (poor)	38%; 12/31 (6 absent; 6 poor)	100%; 7/7 (poor)	NA	+
Abnormal sleep pattern	57%; 4/7	70%; 16/23	75%; 3/4	22%; 2/9	+
Inappropriate laughing	43%; 3/7	43%; 9/21	100%; 4/4	11%; 1/9	+
Bruxism	71%; 5/7	88%; 14/16	89%; 8/9	NA	−
Strabismus	50%; 4/8	95%; 19/20	88%; 7/8	11%; 1/9	+
Epilepsy	75%; 9/12	81%; 29/36	90%; 9/10	56%; 5/9	+
Stereotypic movements	75%; 6/8	85%; 23/27	100%; 7/7	22%; 2/9	+
Feeding difficulties	75%; 6/8	100%; 21/21	100%; 8/8	NA	+
Corpus callosum anomalies	33%; 3/9	83%; 20/24	90%; 9/10 (hypogenesis)	NA	−^b^

*HC* head circumference, *SD* standard deviation, *NA* not available, *cs* conserve site 1, + phenotype observed; − phenotype not observed. Data are displayed as observed percentages and fractions of clinical, neurological, and behavioral anomalies

Balanced translocations, microdeletions, and duplications involving the *FOXG1* gene presented in the Mehrjouy et al. paper [11] include three original cases and the remaining reviewed from the literature

^a^A diagnostic algorithm is proposed to diagnose *FOXG1* syndrome caused by recurrent structural aberrations disrupting a distant enhancer

^b^MRI at 4 month of age was reportedly unremarkable, but not available for review

This case further supports many of the features common to the *FOXG1* syndrome phenotype. In addition, it confirms that such a phenotype occurs in a case involving a chromosomal structural variant resulting in haploinsufficiency via disruption of *FOXG1* regulatory elements located within a previously defined SRO. It also shows that for patients with a similar phenotype and karyotype consistent with balanced structural variants involving 14q11-q13, reflex metaphase FISH analysis using BAC probes of RP11-829F20 (*FOXG1*) and RP11-840N18 (*SRO*)(Fig. 1d) provides an efficient option for making a final diagnosis. By examining the breakpoints of previously reported *FOXG1* syndrome cases (reviewed by Mehrjouy et al. [11]), we are able to conclude that the proposed FISH analysis would help make a genetic diagnosis for 12/12 cases with balanced translocations and for 4/4 cases with submicroscopic microdeletions. It also suggests that submicroscopic balanced rearrangements, such as subtle inversions or insertions, may cause the disease, but would remain undiagnosed without the reflex FISH. Taken together, FISH and *FOXG1* gene sequencing would identify a majority of disease-causing mutation types (Table 1), including translocation, microdeletion, single nucleotide and small indels, and are therefore recommended as a diagnostic algorithm for *FOXG1* syndrome.

## Data Availability

The raw data of CGH and FISH were available upon request.
